# The Dietary Inflammatory Index and All-Cause, Cardiovascular Disease, and Cancer Mortality in the Multiethnic Cohort Study

**DOI:** 10.3390/nu10121844

**Published:** 2018-12-01

**Authors:** Song-Yi Park, Minji Kang, Lynne R. Wilkens, Yurii B. Shvetsov, Brook E. Harmon, Nitin Shivappa, Michael D. Wirth, James R. Hébert, Christopher A. Haiman, Loïc Le Marchand, Carol J. Boushey

**Affiliations:** 1 Cancer Center, University of Hawaii, Honolulu, HI 96813, USA; mkang@cc.hawaii.edu (M.K.); lynne@cc.hawaii.edu (L.R.W.); yshvetso@cc.hawaii.edu (Y.B.S.); loic@cc.hawaii.edu (L.L.M.); cjboushey@cc.hawaii.edu (C.J.B.); 2 Center for Gendered Innovations in Science and Technology Research (GISTeR), Seoul, Korea; 3 School of Public Health, University of Memphis, Memphis, TN 38152, USA; bharmon1@memphis.edu; 4 Cancer Prevention and Control Program, Arnold School of Public Health, University of South Carolina, Columbia, SC 292008, USA; shivappa@mailbox.sc.edu (N.S.); wirthm@mailbox.sc.edu (M.D.W.); jhebert@mailbox.sc.edu (J.R.H.); 5 Norris Comprehensive Cancer Center, University of Southern California, Los Angeles, CA 90033, USA; christopher.haiman@med.usc.edu

**Keywords:** cancer, cardiovascular diseases, cohort, diet, dietary inflammatory index, mortality, multiethnic population

## Abstract

Diet quality based on inflammatory potential, assessed by the Dietary Inflammatory Index (DII^®^), has been related to mortality, but studies from racially/ethnically diverse populations are scarce. Using data from the Multiethnic Cohort Study in Hawaii and California, we investigated the association of the DII with all-cause, cardiovascular disease (CVD) and cancer mortality, both overall and by race/ethnicity. The analysis included 150,405 African Americans, Native Hawaiians, Japanese Americans, Latinos, and Whites aged 45–75 years, with 47,436 deaths during an average follow-up of 18.2 ± 4.9 years. In multivariable-adjusted Cox models, the hazard ratios (95% confidence intervals) for the highest vs. lowest quintile of the DII in men and women were 1.15 (1.09–1.21) and 1.22 (1.14–1.28) for all-cause, 1.13 (1.03–1.23) and 1.29 (1.17–1.42) for CVD, and 1.10 (1.00–1.21) and 1.13 (1.02–1.26) for cancer mortality. In men, an increased risk of all-cause mortality with higher DII scores was found in all racial/ethnic groups except for Native Hawaiians (*P* for heterogeneity < 0.001). Similarly, in women, an increased risk of CVD mortality was found in the four racial/ethnic groups, but not in Native Hawaiians. These findings support the association of a pro-inflammatory diet with a higher risk of mortality and suggest the association may vary by race/ethnicity.

## 1. Introduction

Chronic inflammation provides a substrate for critical mechanisms involved in major diseases, including cardiovascular diseases (CVD) and cancers [1]. Many dietary components are known to be involved in inflammatory processes, including those influencing inflammatory markers [2]. Therefore, dietary components with pro- or anti-inflammatory properties have been examined with respect to their associations with disease outcomes [3,4]. However, diet is a complex of various components that often interact and whose cumulative effect may modify both inflammatory responses and disease outcomes [5]. Thus, the Dietary Inflammatory Index (DII^®^) was developed to assess the inflammatory potential of an individual’s overall diet, based on literature review and scoring of peer-reviewed papers published from 1950–2010 that examined the associations between various dietary components and inflammation [6]. Several dietary indexes have been developed and used to assess diet quality, but only the DII focuses on diet’s effects on inflammation [7]. DII scores have been linked to risk of disease and mortality [8,9,10,11,12,13]. In a recent meta-analysis, a higher DII score, indicating a pro-inflammatory diet, was associated with an increased risk of all-cause mortality [11]. However, data from racially/ethnically-diverse populations are scarce.

The Multiethnic Cohort (MEC) consists of participants mostly from five race/ethnicity groups: African Americans, Native Hawaiians, Japanese Americans, Latinos, and Whites. The MEC thus provides a unique opportunity to examine racial/ethnic differences in risk factors for chronic disease and mortality. In the present study, we examined the association of inflammatory potential of diet, measured by the DII, with mortality from all causes, CVD, and cancer, and whether or not the association varied by race/ethnicity. 

## 2. Materials and Methods

### 2.1. Study Population

The MEC was established to study dietary, lifestyle, and genetic factors in relation to cancer and other chronic diseases [14]. In 1993–1996, more than 215,000 participants aged 45–75 years at recruitment entered the cohort by completing a self-administered, 26-page mailed questionnaire and consenting to participate in the study. They were mostly African Americans, Native Hawaiians, Japanese Americans, Latinos, and Whites living in Hawaii and California. In the current analyses, we excluded participants who did not self-identify as one of the five major racial/ethnic groups (*n* = 13,987) or who reported implausible diets based on total energy intake or its components (*n* = 8241) [15]. Specifically, we calculated a robust standard deviation (RSD) of energy intake, using the truncated normal distribution after excluding the top and bottom 10% tails. Then, all energy values beyond mean ± 3 RSD were excluded. A similar procedure was performed for fat, protein, or carbohydrate intakes to exclude individuals outside the range of mean ± 3.5 RSD. We further excluded participants who had a previous history of cancer, heart attack, angina, or stroke at baseline (*n* = 36,982), or who were missing smoking information (*n* = 6033). Data from a total of 150,405 participants (67,351 men and 83,054 women) remained in the analytic dataset. The institutional review boards at the University of Hawaii and the University of Southern California approved the study protocol.

### 2.2. Dietary Assessment and Covariates

The baseline questionnaire included a quantitative food frequency questionnaire (QFFQ) with more than 180 food items, which was developed using 3-day measured food records completed by approximately 60 men and women from each of the main racial/ethnic groups [14]. The QFFQ enquired on usual intake during the last year by providing eight or nine frequency categories and three usual serving sizes. A calibration study showed satisfactory correlations between three 24-h recalls and the QFFQ from approximately 260 participants in each racial/ethnic-sex group [15]. On the baseline questionnaire, study participants also provided information on socio-demographic factors, other personal behaviors including smoking and physical activity, history of medical conditions, and use of medication.

### 2.3. Dietary Inflammatory Index

The DII was developed and validated as previously described [6,16]. The DII calculation for the MEC also has been described elsewhere [13]. In brief, to determine an inflammatory effect score, nearly 2000 peer-reviewed articles published through 2010 on the association between diet and six inflammatory markers (i.e., C-reactive protein, interleukin (IL)-1β, IL-4, IL-6, IL-10, and tumor necrosis factor (TNF)-α) were reviewed and scored. A total of 45 food components were identified as having a sufficiently robust literature linking to at least one of the six markers. Twenty-eight of the 45 food components were included in the DII calculation for the MEC (Supplementary Table S1).

The DII was standardized to its current range with the use of dietary intake from surveys or studies conducted in 11 countries. A z-score was created for each food component for each participant and then converted to a centered proportion score. DII calculations are based on the density of each food component (intake per 1000 kcal), also known as the energy-adjusted DII (E-DII). A higher DII score indicates a more pro-inflammatory diet and a lower score indicates a more anti-inflammatory diet.

### 2.4. Outcome Ascertainment

Deaths of cohort participants were identified by linkage to the death certificate files in Hawaii and California and to the U.S. National Death Index through December 31, 2014. Deaths from CVD were classified as International Classification of Diseases, Ninth Revision (ICD-9) codes 390-448 or Tenth Revision (ICD-10) codes I00-I78 (major cardiovascular diseases). Deaths from cancer were defined as ICD-9 codes 140-208 or ICD-10 codes C00-C97 (malignant neoplasms). During an average of 18.2 ± 4.9 years of follow-up, we identified 47,436 deaths, including 16,212 from CVD and 13,898 from cancer among 150,405 eligible participants.

### 2.5. Statistical Analysis

A Cox proportional hazards model, with age as the time metric, was used to estimate hazard ratios (HRs) and 95% confidence intervals (CIs) of mortality according to the DII in men and women separately. The DII scores were categorized into quintiles based on the distribution in the entire cohort. The lowest quintile served as a reference category. Trend tests for linearity were performed by including sex- and racial/ethnic-specific medians within each quintile as a continuous variable. The DII also was modeled as a continuous variable to estimate HRs of mortality per one-point increase in the DII score. Basic models were adjusted for age at cohort entry (<50, 50–54, 55–59, 60–64, 65–69, ≥70 years) and race/ethnicity as strata variables. For multivariable-adjusted models, we used a comprehensive smoking model developed for lung cancer studies [17], which included smoking status (never, former, current), average number of cigarettes, squared average number of cigarettes, number of years smoked (time-dependent), number of years since quitting (time-dependent), and interactions of race/ethnicity with smoking status, with average number of cigarettes, with squared average number of cigarettes and with number of years smoked. We further adjusted for body mass index (<25, 25–29.9, ≥30 kg/m^2^), history of diabetes (yes, no), education (≤12, 13–15, ≥16 years), marital status (married, not married), moderate-to-vigorous physical activity (<0.36, 0.36–0.82, 0.38–1.67, ≥1.68 h/day for men; <0.35, 0.35–0.70, 0.71–1.20, ≥1.21 h/day for women), alcohol intake (0, 0.1–5.1, 5.2–22, ≥23 g/day for men; 1, 0.1–2.4, 2.5–9.9, ≥10 g/day for women) and menopausal hormone therapy use (never, former, current) for women only as strata variables and total energy intake (log transformed kcal) as a covariate. Five covariates had missing values: body mass index (*n* = 1549), education (*n* = 634), marital status (*n* = 1131), moderate-to-vigorous physical activity (*n* = 2397), and menopausal hormone therapy use for women (*n* = 169). For missing values, we used multiple imputation models with five iterations, assuming the missing data were missing completely at random, conditional on sex, age, and ethnicity [18]. The analyses were also rerun using a complete case approach which excluded participants with missing data on any of the covariates (*n* = 7090). The results of the multiple imputation models and complete cases were very similar. We present the imputation results in the main tables and in Supplementary Table S2. The complete cases analysis is available in Supplementary Table S3. The proportional hazards assumption was verified by Schoenfeld residuals [19]. We also ran the models for each race/ethnicity separately. Tests for heterogeneity by sex and race/ethnicity were based on the likelihood ratio test. To compare the association with the DII between CVD and cancer mortality, a competing risk analysis was performed with each cause simultaneously modeled as a different event [19]. In sensitivity analyses, we removed deaths occurring within the first three years of follow-up. All analyses were performed using SAS statistical software version 9.4 (SAS Institute Inc., Cary, NC, USA).

## 3. Results

### 3.1. Participant Characteritics

Baseline characteristics of participants are presented by quintile of the DII in Table 1. Compared with men and women in the lowest quintile of the DII (most anti-inflammatory diet), those in the highest quintile (most pro-inflammatory diet) tended to be younger, Native Hawaiian, and current smokers, and to have a higher body mass index, higher total energy intake, and consume more alcohol among drinkers. They were also less likely to have a history of diabetes and to have graduated from college. Men with a higher DII score tended to be less physically active. Women with a higher DII score were less likely to be ever users of menopausal hormone therapy. Median DII scores by sex and race/ethnicity are in Appendix A, Table A1. Men had a higher median score than women across all racial/ethnic groups. Native Hawaiians had the highest score in both men and women, while White men and women and Japanese-American women had the lowest scores within each sex.

### 3.2. DII and Mortality

Higher DII scores were associated with an increased risk of all-cause, CVD, and cancer mortality in both men and women, as either a categorical or continuous variable, when adjusting for age and race/ethnicity (Table 2). After further adjustment for smoking and other covariates, the positive associations attenuated, but remained significant for both men and women. The multivariable-adjusted HR (95% CI) of all-cause mortality was 1.15 (1.09–1.21) in men and 1.21 (1.14–1.28) in women for the highest vs. lowest quintile of the DII. Also, the risk of all-cause mortality increased by 3% per one-point increase in the DII score (HR = 1.03, 95% CI: 1.02–1.04) in both men and women. There was no indication of heterogeneity in the association between men and women (P = 0.83). Similar trends were found for CVD and cancer mortality, although the association was stronger for CVD mortality than for cancer mortality (P for heterogeneity between CVD and cancer mortality <0.001 in both men and women). In the sensitivity analyses excluding deaths occurring within the first three years of follow-up, the findings remained similar (Supplementary Table S2).

### 3.3. DII and Mortaltiy by Race/Ethnicity

In racial/ethnic-specific analyses among men with multivariable adjustment (Table 3), differences in risks of all-cause mortality associated with a higher DII score as both a categorical and a continuous variable were statistically significant (*P* for heterogeneity < 0.001). Latinos and Whites displayed statistically significant increased risks and, in the models with the DII as a continuous variable, African-American and Japanese-American groups also showed a suggestive increase in risk of all-cause mortality. A positive trend for CVD mortality was statistically significant in Japanese-American and White men, although no heterogeneity was found across the five racial/ethnic groups (*P* = 0.24). For cancer mortality, only Latino men showed a significant increase in risk (*P* for heterogeneity = 0.02 across the five racial/ethnic groups and 0.004 between Latinos vs. the other four groups combined).

In women, the associations between the DII and all-cause mortality did not vary across the racial/ethnic groups (*P* for heterogeneity = 0.24), although in Native Hawaiians the HRs for the highest quintile and the linear trend did not reach statistical significance (Table 4). For CVD mortality, Native Hawaiian women did not show an increased risk with higher DII scores either in the categorical or continuous model (*P* for heterogeneity = 0.006 across the five racial/ethnic groups and 0.006 between Native Hawaiians vs. the other four groups combined). No heterogeneity by race/ethnicity was found for cancer mortality in women (*P* = 0.44).

## 4. Discussion

In this large prospective cohort of multiethnic adults, we found that a more pro-inflammatory diet, as measured by the DII, was associated with a higher risk of all-cause, CVD, and cancer mortality in both men and women. Overall, the positive association appeared to be weaker in Native Hawaiians compared with the other four racial/ethnic groups.

Several recent meta-analyses have reported on the relationship between the DII scores and mortality. A meta-analysis of 12 studies found a 23% higher risk of all-cause mortality comparing the highest vs. lowest DII category [11,20]. Another meta-analysis of six studies reported a 37% increased risk of CVD mortality comparing the highest vs. lowest DII category and a 9% increased risk for each one-point increase in the DII score [12]. In a meta-analysis for cancer outcomes, a 67% increase in risk of cancer mortality was found [10]. A randomized controlled trial with low-dose antioxidants reported an increased risk of all-cause and cancer mortality with higher DII scores in the placebo group, but not in the antioxidant-supplemented group [21]. A recent study from the Melbourne Collaborative Cohort Study also found an increased risk of total and CVD mortality with higher DII, by 16% and 30%, respectively [22]. Therefore, all recent reports, including the current analysis, support the association between a pro-inflammatory diet and a higher risk of mortality.

In a recent meta-analysis, an increased risk of CVD (either risk of incident disease or mortality) with higher DII scores was significant only in women and in studies conducted in Europe and North America, but not in men and studies conducted in Australia [12]; although the number of studies in each subgroup was relatively small. The present study also supports a stronger association in women (HR = 1.29 for the highest vs. lowest quintile, 95% CI: 1.17–1.42, *P* for trend < 0.001) than in men (HR = 1.13, 95% CI: 1.03–1.23, *P* for trend = 0.003) for CVD mortality, although the test for heterogeneity by sex was not statistically significant.

In the current study, an increased risk of all-cause mortality with higher DII scores was found in all racial/ethnic groups except Native Hawaiians, among whom the association was not statistically significant and for men, was suggestive of an inverse relationship. These findings are consistent with a previous report from the MEC that examined four diet quality indexes in relation to all-cause mortality [23]. In that study, the four indexes (higher score indicating higher quality diet) were all inversely associated with risk of mortality from all causes, CVD, and cancer in both men and women. Also, the inverse association with all-cause mortality was seen in all racial/ethnic groups except for Native Hawaiians [23]. Native Hawaiians are unique in some aspects of their health profile from other racial/ethnic groups in Hawaii. They are documented as having the highest prevalence of obesity [24], the highest smoking rate in adults [25], the highest cancer mortality [26], and the shortest life expectancy [27]. In the MEC, Native Hawaiians’ diets were lower in quality (measured by the four diet quality indexes) [23] and more pro-inflammatory (measured by the DII), compared with the other racial/ethnic groups. Although Native Hawaiians showed no association of the DII and other diet quality indexes with mortality, the diet quality of Native Hawaiians needs improvement, given their health profile. 

The strengths of this study include the population-based prospective design; the long follow-up period; the large sample size with participants from various racial/ethnic backgrounds; the comprehensive, validated food frequency questionnaire; and a wide range of covariates for diet-mortality analyses. However, there are several limitations to be considered. The current analyses of the DII and mortality were based on a single assessment of diet at baseline. We plan to compute the DII scores for a 10-year follow-up survey, where the QFFQ would be repeated among 45% of the participants, and will be able to compare DII changes over time, as was done in the Women’s Health Initiative Observational Study and Dietary Modification trial control group [28,29]. Despite the comprehensive information on lifestyle factors and careful adjustment for covariates, there is still the possibility of residual confounding by unmeasured or incompletely controlled variables that might be related to both diet and mortality. To compute the DII scores for the MEC, only 28 of the 45 food components originally included for the DII development were used. DII scores that were computed from fewer components, however, have been demonstrated to well predict inflammatory markers and health outcomes in several studies [16,30,31] including the previous report from the MEC [13].

## 5. Conclusions

In summary, these examinations confirm that a more pro-inflammatory diet was associated with a higher risk of all-cause, CVD, and cancer mortality in both men and women. In addition, the association was found in African Americans, Japanese Americans, Latinos, and Whites, but not in Native Hawaiians, the diets of whom were more pro-inflammatory compared with the other racial/ethnic groups.

## Figures and Tables

**Table 1:** Baseline characteristics of participants (*n* = 150,405) in the Multiethnic Cohort by quintiles of Dietary Inflammatory Index (DII) scores.

Characteristics	Quintile 1 (−6.64 to−3.91)	Quintile 2 (−3.90 to −2.89)	Quintile 3 (−2.88 to −1.67)	Quintile 4 (−1.66 to −0.07)	Quintile 5 (−0.06 to +4.95)
**Men (*n* = 67,351)**					
DII score, median (IQR) ^1^	−4.43 (0.63)	−3.38 (0.51)	−2.28 (0.60)	−0.90 (0.79)	1.09 (1.36)
Age at cohort entry, years, median (IQR)	62.0 (14.0)	61.0 (14.0)	60.0 (14.0)	59.0 (15.0)	56.0 (14.0)
Race/ethnicity, *n* (% column)					
African American	1044 (12.1)	1289 (12.4)	1653 (12.7)	1945 (12.2)	2517 (13.0)
Native Hawaiian	494 (5.7)	548 (5.3)	746 (5.7)	1137 (7.1)	1938 (10.0)
Japanese American	2795 (32.5)	2760 (26.5)	3510 (27.0)	4808 (30.1)	6873 (35.5)
Latino	1824 (21.2)	2792 (26.8)	3734 (28.7)	4405 (27.6)	3635 (18.8)
White	2438 (28.4)	3010 (28.9)	3368 (25.9)	3665 (23.0)	4423 (22.8)
Body mass index ^2^, kg/m^2^, median (IQR)	25.6 (4.4)	25.9 (4.5)	26.1 (4.7)	26.3 (4.7)	26.2 (5.2)
Physical activity ^2,3^, h/day, median (IQR)	1.07 (1.46)	0.93 (1.32)	0.82 (1.32)	0.82 (1.21)	0.82 (1.21)
History of diabetes, *n* (%)	1333 (15.5)	1272 (12.2)	1585 (12.2)	1646 (10.3)	1386 (7.1)
Smoking status, *n* (% column)					
Never	3337 (38.8)	3925 (37.7)	4507 (34.6)	4959 (31.1)	4918 (25.4)
Former	4552 (53.0)	5323 (51.2)	6583 (50.6)	7862 (49.3)	8633 (44.5)
Current	706 (8.2)	1151 (11.1)	1921 (14.8)	3139 (19.7)	5835 (30.1)
Education ^2^, *n* (% column)					
≤12 years	3104 (36.1)	3861 (37.1)	5192 (39.9)	6602 (41.4)	7956 (41.0)
13–15 years	2413 (28.1)	2862 (27.5)	3594 (27.6)	4681 (29.3)	6276 (32.4)
≥16 years	3078 (35.8)	3676 (35.3)	4225 (32.5)	4677 (29.3)	5154 (26.6)
Married ^2^, *n* (%)	6622 (77.0)	8111 (78.0)	10,154 (78.0)	12,370 (77.5)	14,387 (74.2)
Energy intake, kcal/day, median (IQR)	2094 (1195)	2139 (1237)	2186 (1288)	2231 (1293)	2340 (1380)
Alcohol intake ^4^, g/day, median (IQR)	9.1 (19.0)	8.2 (19.1)	9.5 (21.3)	11.7 (27.6)	16.4 (39.6)
**Women (*n* = 83,054)**					
DII score, median (IQR)	−4.48 (0.66)	−3.42 (0.51)	−2.34 (0.60)	−0.97 (0.79)	0.92 (1.32)
Age at cohort entry, years, median (IQR)	62.0 (14.0)	60.0 (14.0)	59.0 (15.0)	56.0 (14.0)	54.0 (14.0)
Race/ethnicity, *n* (% column)					
African American	3613 (17.4)	3392 (17.8)	3044 (18.1)	2705 (18.6)	2446 (20.6)
Native Hawaiian	1328 (6.4)	1135 (6.0)	1069 (6.4)	1226 (8.4)	1397 (11.7)
Japanese American	7062 (34.0)	5181 (27.2)	4689 (27.9)	4016 (27.7)	3256 (27.4)
Latino	3779 (18.2)	4303 (22.6)	3943 (23.4)	3285 (22.6)	2025 (17.0)
White	4978 (24.0)	5041 (26.5)	4081 (24.3)	3292 (22.7)	2768 (23.3)
Body mass index ^2^, kg/m^2^, median (IQR)	24.8 (6.3)	25.1 (6.2)	25.4 (6.7)	25.8 (7.0)	26.0 (7.6)
Physical activity ^2,3^, h/day, median (IQR)	0.71 (1.07)	0.71 (1.07)	0.71 (0.86)	0.71 (0.86)	0.71 (0.86)
History of diabetes, *n* (%)	2358 (11.4)	1806 (9.5)	1480 (8.8)	1230 (8.5)	827 (7.0)
Smoking status, *n* (% column)					
Never	13,115 (63.2)	11,646 (61.1)	9843 (58.5)	7979 (54.9)	5674 (47.7)
Former	5886 (28.4)	5473 (28.7)	4697 (27.9)	3930 (27.1)	2917 (24.5)
Current	1759 (8.5)	1933 (10.1)	2286 (13.6)	2615 (18.0)	3301 (27.8)
Education ^2^, *n* (% column)					
≤12 years	8834 (42.6)	8247 (43.3)	7733 (46.0)	6736 (46.4)	5637 (47.4)
13–15 years	6056 (29.2)	5674 (29.8)	4924 (29.3)	4493 (30.9)	3777 (31.8)
≥16 years	5870 (28.3)	5131 (26.9)	4169 (24.8)	3295 (22.7)	2478 (20.8)
Married ^2^, *n* (%)	12,577 (60.6)	11,585 (60.8)	10,214 (60.7)	8820 (60.7)	6985 (58.7)
MHT ^2,5^ ever use, *n* (%)	13,849 (66.7)	12,973 (68.1)	11,753 (69.9)	10,464 (72.0)	8977 (75.5)
Energy intake, kcal/day, median (IQR)	1745 (991)	1753 (1023)	1760 (1039)	1772 (1086)	1869 (1183)
Alcohol intake ^4^, g/day, median (IQR)	3.5 (10.6)	3.0 (9.0)	3.1 (10.1)	3.8 (12.3)	4.5 (19.9)

^1^ IQR, interquartile range. ^2^ Missing values were imputed using multiple imputation. ^3^ Moderate-to-vigorous physical activity. ^4^ Among drinkers. ^5^ MHT, menopausal hormone therapy.

**Table 2:** Hazard ratios (HR) (95% confidence intervals (CI)) for all-causes, cardiovascular disease (CVD), and cancer mortality according to quintiles of Dietary Inflammatory Index scores in the Multiethnic Cohort, 1993–2014.

	Men (*n* = 67,351)			Women (*n* = 83,054)			*P* for Heterogeneity ^3^
	Deaths *n*	HR (95% CI) ^1^	HR (95% CI) ^2^	Deaths *n*	HR (95% CI) ^1^	HR (95% CI) ^2^	
**All Causes**							
Quintile 1	3308	1.00	1.00	6181	1.00	1.00	
Quintile 2	3832	1.02 (0.97–1.07)	1.03 (0.98–1.09)	5399	1.03 (1.00–1.07)	1.04 (0.99–1.09)	
Quintile 3	4797	1.09 (1.04–1.14)	1.03 (0.98–1.09)	4578	1.10 (1.06–1.14)	1.06 (1.01–1.12)	
Quintile 4	5632	1.16 (1.11–1.21)	1.07 (1.01–1.12)	3764	1.20 (1.15–1.25)	1.12 (1.06–1.18)	
Quintile 5	6822	1.42 (1.36–1.48)	1.15 (1.09–1.21)	3123	1.45 (1.39–1.51)	1.21 (1.14–1.28)	
*P* for trend ^4^		<0.001	<0.001		<0.001	<0.001	0.83
Continuous		1.07 (1.06–1.08)	1.03 (1.02–1.04)		1.07 (1.06–1.07)	1.03 (1.02–1.04)	
**CVD**							
Quintile 1	1176	1.00	1.00	2116	1.00	1.00	
Quintile 2	1342	1.01 (0.93–1.09)	1.04 (0.95–1.14)	1834	1.04 (0.97–1.10)	1.06 (0.98–1.15)	
Quintile 3	1721	1.10 (1.02–1.18)	1.04 (0.95–1.13)	1609	1.16 (1.09–1.24)	1.12 (1.04–1.22)	
Quintile 4	1961	1.15 (1.07–1.23)	1.09 (1.00–1.19)	1245	1.22 (1.13–1.31)	1.11 (1.02–1.22)	
Quintile 5	2201	1.31 (1.22–1.40)	1.13 (1.03–1.23)	1007	1.47 (1.36–1.58)	1.29 (1.17–1.42)	
*P* for trend ^4^		<0.001	0.003		<0.001	<0.001	0.78
Continuous		1.05 (1.04–1.07)	1.03 (1.01–1.04)		1.07 (1.05–1.08)	1.04 (1.03–1.06)	
**Cancer**							
Quintile 1	906	1.00	1.00	1658	1.00	1.00	
Quintile 2	1098	1.05 (0.97–1.15)	1.02 (0.92–1.13)	1481	1.02 (0.95–1.09)	1.02 (0.94–1.12)	
Quintile 3	1390	1.12 (1.03–1.22)	1.01 (0.92–1.12)	1263	1.05 (0.98–1.13)	1.01 (0.92–1.10)	
Quintile 4	1690	1.22 (1.12–1.32)	1.01 (0.92–1.11)	1134	1.20 (1.11–1.29)	1.06 (0.97–1.17)	
Quintile 5	2273	1.58 (1.46–1.70)	1.10 (1.00–1.21)	1005	1.45 (1.34–1.57)	1.13 (1.02–1.26)	
*P* for trend ^4^		<0.001	0.03		<0.001	0.02	0.92
Continuous		1.09 (1.08–1.10)	1.02 (1.01–1.04)		1.07 (1.05–1.08)	1.02 (1.00–1.04)	

^1^ Adjusted for age at cohort entry and race/ethnicity. ^2^ Adjusted for age at cohort entry, race/ethnicity, body mass index, history of diabetes, education, marital status, physical activity, alcohol intake, energy intake, and menopausal hormone therapy use (for women only) in the smoking model including smoking status, average number of cigarettes, squared average number of cigarettes, number of years smoked (time-dependent), number of years since quitting (time-dependent), and interactions between ethnicity and smoking status, average number of cigarettes, squared average number of cigarettes and number of years smoked. ^3^ Based on the multivariable-adjusted models. ^4^ Tests for linearity.

**Table 3:** Hazard ratios (HR) (95% confidence intervals (CI)) for all-causes, cardiovascular disease (CVD), and cancer mortality according to quintiles of Dietary Inflammatory Index scores by race/ethnicity among men in the Multiethnic Cohort, 1993–2014.

	African American		Native Hawaiian		Japanese American		Latino		White		*P* for Heterogeneity
	Deaths *n*	HR (95% CI) ^1^	Deaths *n*	HR (95% CI) ^1^	Deaths *n*	HR (95% CI) ^1^	Deaths *n*	HR (95% CI) ^1^	Deaths *n*	HR (95% CI) ^1^	
**All Causes**											
Quintile 1	548	1.00	213	1.00	1139	1.00	624	1.00	784	1.00	
Quintile 2	677	1.15 (0.99–1.33)	219	0.89 (0.67–1.18)	1075	1.01 (0.92–1.11)	942	1.10 (0.98–1.23)	919	0.98 (0.88–1.10)	
Quintile 3	822	1.07 (0.93–1.23)	292	0.91 (0.70–1.19)	1318	0.98 (0.89–1.07)	1257	1.11 (0.99–1.24)	1108	1.07 (0.96–1.19)	
Quintile 4	969	1.12 (0.98–1.28)	397	0.81 (0.63–1.04)	1546	0.99 (0.91–1.08)	1498	1.18 (1.05–1.31)	1222	1.11 (1.00–1.24)	
Quintile 5	1290	1.13 (0.99–1.30)	653	0.85 (0.67–1.07)	1987	1.06 (0.97–1.16)	1243	1.32 (1.18–1.48)	1649	1.24 (1.11–1.38)	
*P* for trend ^2^		0.20		0.20		0.15		<0.001		<0.001	<0.001
Continuous		1.02 (1.00–1.04)		0.98 (0.94–1.01)		1.01 (1.00–1.02)		1.05 (1.03–1.07)		1.05 (1.03–1.06)	
**CVD**											
Quintile 1	229	1.00	77	1.00	369	1.00	239	1.00	262	1.00	
Quintile 2	247	1.03 (0.82–1.30)	78	0.96 (0.60–1.55)	362	1.03 (0.88–1.21)	354	1.03 (0.85–1.25)	301	1.09 (0.90–1.33)	
Quintile 3	311	0.90 (0.72–1.12)	112	0.96 (0.62–1.50)	436	1.00 (0.86–1.17)	466	1.07 (0.89–1.28)	396	1.17 (0.97–1.41)	
Quintile 4	367	0.98 (0.79–1.22)	146	0.88 (0.58–1.34)	498	1.03 (0.88–1.20)	544	1.15 (0.96–1.37)	406	1.27 (1.05–1.53)	
Quintile 5	465	0.97 (0.78–1.21)	240	0.96 (0.64–1.42)	619	1.16 (1.00–1.35)	377	1.08 (0.89–1.31)	500	1.32 (1.09–1.59)	
*P* for trend ^2^		0.82		0.84		0.05		0.26		0.002	0.24
Continuous		1.01 (0.97–1.04)		1.01 (0.95–1.06)		1.03 (1.00–1.05)		1.02 (0.99–1.05)		1.05 (1.02–1.08)	
**Cancer**											
Quintile 1	142	1.00	56	1.00	294	1.00	173	1.00	241	1.00	
Quintile 2	201	1.22 (0.94–1.60)	62	0.94 (0.56–1.58)	307	1.04 (0.87–1.23)	248	1.02 (0.82–1.27)	280	0.93 (0.77–1.13)	
Quintile 3	250	1.19 (0.92–1.53)	94	1.17 (0.72–1.93)	377	0.97 (0.82–1.15)	349	1.05 (0.85–1.29)	320	0.95 (0.79–1.15)	
Quintile 4	298	1.26 (0.98–1.62)	112	0.88 (0.55–1.41)	466	0.93 (0.79–1.09)	427	1.07 (0.88–1.32)	387	0.99 (0.82–1.20)	
Quintile 5	425	1.25 (0.97–1.61)	214	1.08 (0.69–1.66)	678	0.96 (0.82–1.13)	427	1.37 (1.11–1.68)	529	1.06 (0.88–1.28)	
*P* for trend ^2^		0.18		0.73		0.36		<0.001		0.24	0.02
Continuous		1.02 (0.99–1.06)		1.01 (0.95–1.07)		1.00 (0.97–1.02)		1.06 (1.03–1.10)		1.03 (1.00–1.05)	

^1^ Adjusted for age at cohort entry, body mass index, history of diabetes, education, marital status, physical activity, alcohol intake, and energy intake in n the smoking model including smoking status, average number of cigarettes, squared average number of cigarettes, number of years smoked (time-dependent), and number of years since quitting (time-dependent). ^2^ Tests for linearity.

**Table 4:** Hazard ratios (HR) (95% confidence intervals (CI)) for all-causes, cardiovascular disease (CVD), and cancer mortality according to quintiles of Dietary Inflammatory Index scores by race/ethnicity among women in the Multiethnic Cohort, 1993–2014.

	African American		Native Hawaiian		Japanese American		Latino		White		*P* for Heterogeneity
	Deaths *n*	HR (95% CI) ^1^	Deaths *n*	HR (95% CI) ^1^	Deaths *n*	HR (95% CI) ^1^	Deaths *n*	HR (95% CI) ^1^	Deaths *n*	HR (95% CI) ^1^	
**All Causes**											
Quintile 1	1494	1.00	430	1.00	1934	1.00	966	1.00	1357	1.00	
Quintile 2	1381	1.04 (0.95–1.15)	325	1.16 (0.93–1.47)	1359	1.07 (0.99–1.15)	1030	0.95 (0.85–1.06)	1304	1.05 (0.96–1.16)	
Quintile 3	1189	1.08 (0.98–1.19)	289	1.02 (0.80–1.29)	1078	1.03 (0.95–1.12)	945	1.05 (0.94–1.18)	1077	1.11 (1.00–1.23)	
Quintile 4	1030	1.23 (1.10–1.36)	313	1.09 (0.86–1.38)	767	1.07 (0.97–1.17)	774	1.10 (0.98–1.24)	880	1.09 (0.97–1.22)	
Quintile 5	893	1.25 (1.12–1.40)	324	1.16 (0.92–1.48)	565	1.13 (1.01–1.27)	485	1.17 (1.02–1.34)	856	1.30 (1.16–1.47)	
*P* for trend ^2^		<0.001		0.32		0.04		0.003		<0.001	0.24
Continuous		1.04 (1.02–1.06)		1.01 (0.98–1.05)		1.02 (1.00–1.04)		1.04 (1.02–1.06)		1.04 (1.02–1.06)	
**CVD**											
Quintile 1	600	1.00	142	1.00	610	1.00	362	1.00	402	1.00	
Quintile 2	540	1.06 (0.92–1.23)	108	1.11 (0.75–1.64)	454	1.12 (0.98–1.28)	360	0.91 (0.76–1.09)	372	1.12 (0.93–1.35)	
Quintile 3	505	1.17 (1.00–1.37)	102	0.98 (0.65–1.48)	352	1.08 (0.93–1.26)	307	0.95 (0.79–1.15)	343	1.35 (1.11–1.64)	
Quintile 4	418	1.27 (1.08–1.50)	94	0.71 (0.46–1.08)	250	1.16 (0.98–1.37)	225	0.87 (0.70–1.06)	258	1.21 (0.97–1.50)	
Quintile 5	331	1.39 (1.16–1.66)	95	0.86 (0.56–1.34)	169	1.14 (0.93–1.39)	162	1.17 (0.93–1.48)	250	1.60 (1.27–2.01)	
*P* for trend ^2^		<0.001		0.18		0.10		0.50		<0.001	0.006
Continuous		1.06 (1.03–1.09)		0.95 (0.88–1.01)		1.02 (0.99–1.05)		1.03 (0.99–1.07)		1.07 (1.03–1.11)	
**Cancer**											
Quintile 1	413	1.00	117	1.00	491	1.00	261	1.00	376	1.00	
Quintile 2	372	0.99 (0.83–1.18)	93	1.20 (0.79–1.82)	349	1.04 (0.89–1.21)	275	0.91 (0.74–1.11)	392	1.11 (0.93–1.32)	
Quintile 3	304	1.00 (0.83–1.21)	78	0.93 (0.60–1.42)	294	0.98 (0.83–1.15)	277	1.01 (0.82–1.24)	310	1.07 (0.89–1.29)	
Quintile 4	306	1.10 (0.90–1.34)	104	1.38 (0.91–2.11)	230	0.96 (0.80–1.15)	244	1.15 (0.93–1.42)	250	0.99 (0.81–1.21)	
Quintile 5	271	1.06 (0.86–1.30)	114	1.47 (0.97–2.23)	188	1.05 (0.85–1.28)	148	1.04 (0.81–1.33)	284	1.30 (1.05–1.60)	
*P* for trend ^2^		0.40		0.05		0.98		0.20		0.09	0.44
Continuous		1.01 (0.98–1.04)		1.06 (1.00–1.13)		1.00 (0.97–1.03)		1.02 (0.98–1.06)		1.03 (1.00–1.06)	

^1^ Adjusted for age at cohort entry, BMI, history of diabetes, education, marital status, physical activity, alcohol intake, energy intake, and menopausal hormone therapy use. In the smoking model including smoking status, average number of cigarettes, squared average number of cigarettes, number of years smoked (time-dependent), and number of years since quitting (time-dependent). ^2^ Tests for linearity.

## Supplementary Materials

The following are available online at http://www.mdpi.com/2072-6643/10/12/1844/s1, Table S1: The 45 food components identified for the Dietary Inflammatory Index calculation; Table S2: Hazard ratios (HR) (95% confidence intervals (CI)) for all-cause, cardiovascular disease (CVD), and cancer mortality according to quintiles of Dietary Inflammatory Index scores excluding deaths occurred within the first three years of follow-up in the Multiethnic Cohort Study, 1993–2014; Table S3: Hazard ratios (HR) (95% confidence intervals (CI)) for all-cause, cardiovascular disease (CVD), and cancer mortality according to quintiles of Dietary Inflammatory Index scores excluding participants with missing values on any of the covariates (complete cases analysis) in the Multiethnic Cohort Study, 1993–2014.

## Appendix A

**Table A1:** Median and interquartile range (IQR) of Dietary Inflammatory Index (DII) scores by sex and race/ethnicity in the Multiethnic Cohort.

Race/Ethnicity	Men		Women	
	*n*	Median (IQR)	*n*	Median (IQR)
African American	8448	−1.49 (3.34)	15,200	−2.67 (2.89)
Native Hawaiian	4863	−0.74 (3.52)	6155	−2.21 (3.43)
Japanese American	20,746	−1.24 (3.51)	24,204	−2.92 (2.83)
Latino	16,390	−1.72 (2.80)	17,335	−2.73 (2.50)
White	16,904	−1.81 (3.31)	20,160	−2.87 (2.64)
Total	67,351	−1.51 (3.30)	83,054	−2.78 (2.77)
