# Supplementary material for: The Dietary Inflammatory Index and All-Cause, Cardiovascular Disease, and Cancer Mortality in the Multiethnic Cohort Study
Source: Nutrients. 2018 Dec 1;10(12):1844. doi: 10.3390/nu10121844 (PMC6315679; doi:10.3390/nu10121844)
Supplement: Supplementary file 1 [file nutrients-10-01844-s001.pdf]

nutrients

MDPI

Article

# The Dietary Inflammatory Index and All-Cause, Cardiovascular Disease, and Cancer Mortality in the Multiethnic Cohort Study

**Song-Yi Park [1,*], Minji Kang [1,2], Lynne R. Wilkens [1], Yurii B. Shvetsov [1], Brook E. Harmon [3], Nitin Shivappa [4], Michael D. Wirth [4], James R. Hébert [4], Christopher A. Haiman [5], Loïc Le Marchand [1] and Carol J. Boushey [1]**

1 University of Hawaii Cancer Center, Honolulu, HI 96813, USA; mkang@cc.hawaii.edu (M.K.); lynne@cc.hawaii.edu (L.R.W.); yshvetso@cc.hawaii.edu (Y.B.S.); loic@cc.hawaii.edu (L.L.M.); cjboushey@cc.hawaii.edu (C.J.B.)
2 Center for Gendered Innovations in Science and Technology Research (GISTeR), Seoul, Korea
3 School of Public Health, University of Memphis, Memphis, TN 38152, USA; bharmon1@memphis.edu
4 Cancer Prevention and Control Program, Arnold School of Public Health, University of South Carolina, Columbia, SC 292008, USA; shivappa@mailbox.sc.edu (N.S.); wirthm@mailbox.sc.edu (M.D.W.); jhebert@mailbox.sc.edu (J.R.H.)
5 University of Southern California Norris Comprehensive Cancer Center, Los Angeles, CA 90033, USA; christopher.haiman@med.usc.edu
* Correspondence: spark@cc.hawaii.edu; Tel.: +1-808-564-5947

## Supplementary Materials

**Supplementary Table S1.** The 45 food components identified for the Dietary Inflammatory Index calculation.

| | |
|---|---|
| 28 components included in the DII calculation for the Multiethnic Cohort | Carbohydrate; protein; total fat; saturated, monounsaturated, and polyunsaturated fats; ω-3 and ω-6 FAs; alcohol; fiber; cholesterol; vitamins A, B-6, B-12, C, D, and E; thiamin; riboflavin; niacin; iron; magnesium; zinc; selenium; folate; β-carotene; isoflavones; and caffeine |
| 17 components not included in the DII calculation for the Multiethnic Cohort | Eugenol; garlic; ginger; onion; trans fat; turmeric; green tea; black tea; falan-3-ol; flavones; flavonols; flavonones; anthocyanins; pepper; thyme; oregano; and rosemary |

**Supplementary Table S2.** Hazard ratios (HR) (95% confidence intervals (CI)) for all-cause, cardiovascular disease (CVD), and cancer mortality according to quintiles of Dietary Inflammatory Index scores excluding deaths occurred within the first three years of follow-up in the Multiethnic Cohort Study, 1993-2014.

| | Men | | | Women | | | *P* for Heterogeneity[3] |
|---|---|---|---|---|---|---|---|
| | Deaths *n* | HR (95% CI)[1] | HR (95% CI)[2] | Deaths *n* | HR (95% CI)[1] | HR (95% CI)[2] | |
| **All causes** | | | | | | | |
| Quintile 1 | 3,115 | 1.00 | 1.00 | 5,930 | 1.00 | 1.00 | |
| Quintile 2 | 3,597 | 1.02 (0.97-1.07) | 1.03 (0.98-1.09) | 5,144 | 1.03 (0.99-1.07) | 1.03 (0.99-1.08) | |
| Quintile 3 | 4,490 | 1.08 (1.03-1.13) | 1.04 (0.98-1.09) | 4,363 | 1.10 (1.06-1.14) | 1.06 (1.01-1.11) | |
| Quintile 4 | 5,249 | 1.16 (1.11-1.21) | 1.07 (1.01-1.12) | 3,559 | 1.19 (1.14-1.24) | 1.11 (1.05-1.17) | |
| Quintile 5 | 6,292 | 1.40 (1.34-1.46) | 1.15 (1.09-1.21) | 2,952 | 1.44 (1.38-1.51) | 1.21 (1.14-1.28) | |
| P for trend | | <0.001 | <0.001 | | <0.001 | <0.001 | 0.77 |
| Continuous | | 1.07 (1.06-1.07) | 1.03 (1.02-1.03) | | 1.06 (1.06-1.07) | 1.03 (1.02-1.04) | |
| **CVD** | | | | | | | |
| Quintile 1 | 1,100 | 1.00 | 1.00 | 2,026 | 1.00 | 1.00 | |
| Quintile 2 | 1,254 | 1.01 (0.93-1.09) | 1.04 (0.95-1.15) | 1,751 | 1.03 (0.97-1.10) | 1.07 (0.99-1.16) | |
| Quintile 3 | 1,621 | 1.11 (1.03-1.20) | 1.05 (0.96-1.15) | 1,539 | 1.16 (1.09-1.24) | 1.13 (1.04-1.23) | |
| Quintile 4 | 1,806 | 1.13 (1.05-1.22) | 1.08 (0.99-1.19) | 1,168 | 1.20 (1.11-1.29) | 1.10 (1.00-1.20) | |
| Quintile 5 | 2,021 | 1.30 (1.20-1.40) | 1.14 (1.03-1.25) | 960 | 1.48 (1.36-1.60) | 1.29 (1.17-1.43) | |
| P for trend | | <0.001 | 0.005 | | <0.001 | <0.001 | 0.73 |
| Continuous | | 1.05 (1.04-1.06) | 1.02 (1.01-1.04) | | 1.07 (1.05-1.08) | 1.04 (1.02-1.06) | |
| **Cancer** | | | | | | | |
| Quintile 1 | 841 | 1.00 | 1.00 | 1,562 | 1.00 | 1.00 | |
| Quintile 2 | 1,024 | 1.06 (0.97-1.16) | 1.02 (0.92-1.14) | 1,378 | 1.01 (0.94-1.08) | 1.01 (0.92-1.10) | |
| Quintile 3 | 1,287 | 1.12 (1.03-1.22) | 1.02 (0.92-1.13) | 1,191 | 1.05 (0.98-1.14) | 1.01 (0.92-1.11) | |
| Quintile 4 | 1,564 | 1.21 (1.11-1.32) | 1.01 (0.92-1.12) | 1,066 | 1.19 (1.10-1.29) | 1.06 (0.96-1.17) | |
| Quintile 5 | 2,093 | 1.56 (1.44-1.69) | 1.11 (1.00-1.22) | 941 | 1.44 (1.33-1.57) | 1.13 (1.02-1.26) | |
| P for trend | | <0.001 | 0.04 | | <0.001 | 0.02 | 0.90 |
| Continuous | | 1.09 (1.07-1.10) | 1.02 (1.01-1.04) | | 1.07 (1.05-1.08) | 1.02 (1.00-1.04) | |

[1] Adjusted for age at cohort entry and race/ethnicity. [2] Adjusted for age at cohort entry, race/ethnicity, body mass index, history of diabetes, education, marital status, physical activity, alcohol intake, energy intake, and menopausal hormone therapy use (for women only) in the smoking model including smoking status, average number of cigarettes, squared average number of cigarettes, number of years smoked (time-dependent), number of years since quitting (time-dependent), and interactions between ethnicity and smoking status, average number of cigarettes, squared average number of cigarettes and number of years smoked. [3] Based on the multivariable-adjusted models.

**Supplementary Table S3.** Hazard ratios (HR) (95% confidence intervals (CI)) for all-cause, cardiovascular disease (CVD), and cancer mortality according to quintiles of Dietary Inflammatory Index scores excluding participants with missing values on any of the covariates (complete cases analysis) in the Multiethnic Cohort Study, 1993-2014.

| | Men (n=65,605) | | | Women (n=77,710) | | | *P* for Heterogeneity[3] |
|---|---|---|---|---|---|---|---|
| | Deaths *n* | HR (95% CI)[1] | HR (95% CI)[2] | Deaths *n* | HR (95% CI)[1] | HR (95% CI)[2] | |
| **All causes** | | | | | | | |
| Quintile 1 | 3195 | 1.00 | 1.00 | 5591 | 1.00 | 1.00 | |
| Quintile 2 | 3692 | 1.02 (0.97-1.07) | 1.03 (0.97-1.09) | 4915 | 1.04 (1.00-1.08) | 1.04 (0.99-1.10) | |
| Quintile 3 | 4629 | 1.09 (1.04-1.14) | 1.03 (0.97-1.09) | 4116 | 1.10 (1.06-1.15) | 1.06 (1.01-1.12) | |
| Quintile 4 | 5430 | 1.16 (1.11-1.22) | 1.07 (1.01-1.12) | 3392 | 1.20 (1.15-1.25) | 1.10 (1.04-1.16) | |
| Quintile 5 | 6571 | 1.41 (1.36-1.48) | 1.15 (1.09-1.21) | 2828 | 1.46 (1.39-1.53) | 1.21 (1.14-1.28) | |
| P for trend | | <0.001 | <0.001 | | <0.001 | <0.001 | 0.90 |
| Continuous | | 1.07 (1.06-1.08) | 1.03 (1.02-1.04) | | 1.07 (1.06-1.07) | 1.03 (1.02-1.04) | |
| **CVD** | | | | | | | |
| Quintile 1 | 1125 | 1.00 | 1.00 | 1886 | 1.00 | 1.00 | |
| Quintile 2 | 1298 | 1.02 (0.94-1.10) | 1.05 (0.95-1.15) | 1655 | 1.05 (0.98-1.12) | 1.08 (0.99-1.17) | |
| Quintile 3 | 1666 | 1.11 (1.03-1.20) | 1.05 (0.96-1.15) | 1417 | 1.16 (1.08-1.24) | 1.13 (1.03-1.23) | |
| Quintile 4 | 1887 | 1.15 (1.07-1.24) | 1.11 (1.01-1.21) | 1106 | 1.22 (1.13-1.31) | 1.11 (1.00-1.22) | |
| Quintile 5 | 2115 | 1.32 (1.22-1.42) | 1.14 (1.04-1.25) | 894 | 1.48 (1.36-1.60) | 1.29 (1.16-1.44) | |
| P for trend | | <0.001 | 0.002 | | <0.001 | <0.001 | 0.93 |
| Continuous | | 1.05 (1.04-1.07) | 1.03 (1.01-1.04) | | 1.07 (1.05-1.08) | 1.04 (1.02-1.06) | |
| **Cancer** | | | | | | | |
| Quintile 1 | 878 | 1.00 | 1.00 | 1527 | 1.00 | 1.00 | |
| Quintile 2 | 1051 | 1.04 (0.95-1.14) | 1.00 (0.90-1.11) | 1381 | 1.03 (0.96-1.11) | 1.04 (0.95-1.13) | |
| Quintile 3 | 1350 | 1.13 (1.04-1.23) | 1.01 (0.91-1.11) | 1165 | 1.06 (0.98-1.14) | 1.01 (0.92-1.11) | |
| Quintile 4 | 1644 | 1.22 (1.13-1.33) | 1.01 (0.92-1.11) | 1052 | 1.21 (1.11-1.31) | 1.04 (0.94-1.15) | |
| Quintile 5 | 2199 | 1.57 (1.45-1.70) | 1.09 (0.99-1.20) | 931 | 1.46 (1.35-1.59) | 1.15 (1.03-1.28) | |
| P for trend | | <0.001 | 0.03 | | <0.001 | 0.03 | 0.96 |
| Continuous | | 1.09 (1.08-1.10) | 1.02 (1.01-1.04) | | 1.07 (1.05-1.08) | 1.02 (1.00-1.03) | |

[1] Adjusted for age at cohort entry and race/ethnicity. [2] Adjusted for age at cohort entry, race/ethnicity, body mass index, history of diabetes, education, marital status, physical activity, alcohol intake, energy intake, and menopausal hormone therapy use (for women only) in the smoking model including smoking status, average number of cigarettes, squared average number of cigarettes, number of years smoked (time-dependent), number of years since quitting (time-dependent), and interactions between ethnicity and smoking status, average number of cigarettes, squared average number of cigarettes and number of years smoked. [3] Based on the multivariable-adjusted models.
